
JOURNAL INFORMATION
==============================
NLM Title Abbreviation: ScientificWorldJournal
Journal ID: ScientificWorldJournal
NLM Journal ID: 101131163
Journal ID: TSWJ

The Scientific World Journal

ISSN: 2356-6140
EISSN: 1537-744X
Publisher: Wiley

ARTICLE INFORMATION
==============================
PMCID: PMC6083951
PMID: 30147578
DOI: 10.1100/tsw.2001.194
Article ID: 250156
Article version: 1
Subjects: Short Report

Sex Hormones, Apoptosis and the Fas/Fas Ligand System in Normal Endometrial Tissue Remodeling

Mor Gil *
Aschkenazi Sarit
Song Joon
Department of Obstetrics and Gynecology, Yale University School of Medicine, 333 Cedar St., New Haven, Connecticut, USA
*Gil Mor: gil.mor@yale.edu
Publication date: 2001
Electronic publication date: 2001 Nov 8
Volume: 1
First page: 99
Last page: 99
Copyright: Copyright © 2001 Gil Mor et al.
Copyright year: 2001
License: This is an open access article distributed under the Creative Commons Attribution License, which permits unrestricted use, distribution, and reproduction in any medium, provided the original work is properly cited.
License URL: https://creativecommons.org/licenses/by/3.0/

Funding: National Institutes of Health R01 HD37137

==============================
Miami *Nature Biotechnology* Short Reports
*TheScientificWorld* (2001) 1, 99SR
ISSN 1532-2246; DOI 10.1100/tsw.2001.194

### SEX HORMONES, APOPTOSIS AND THE FAS/FAS LIGAND SYSTEM IN NORMAL ENDOMETRIAL TISSUE REMODELING

Gil Mor*, Sarit Aschkenazi, and Joon Song

Department of Obstetrics and Gynecology, Yale University School of Medicine, 333 Cedar St., New Haven, Connecticut, USA

- 1. * gil.mor@yale.edu

**INTRODUCTION.** Tissue remodeling is an important physiological mechanism for maintaining adequate cellular balance in differentiated tissues showing high turnover, and for cancer prevention. The human endometrium is continuously undergoing remodeling mediated by survival and differentiation factors such as estrogen and progesterone. Absence of survival factors at the end of each differentiation cycle results in cellular death of the endometrium, via activation of apoptotic genes such as the Fas/Fas Ligand (FasL) system. In contrast to past studies, and consistent with the above information, new data indicate that menstruation results from activation of apoptotic genes. We hypothesized that remodeling of endometrial tissue is mediated by activation of the Fas/FasL system as result of hormonal changes in the endometrium's microenvironment.

**METHOD.** We performed *in vitro* studies using primary cultures of human endometrial cells and the estrogen and progesterone receptor (ER and PR)-positive Ishikawa endometrial cancer cell line. Cells were incubated with physiologic concentrations of estradiol, progesterone or a combination of both ($10^{-8}$M). The endometrial cells were then incubated in a hormone-depleted milieu and evaluated for apoptosis (MTT assay), Fas and FasL mRNA (RT-PCR) and protein (Western blot) expressions. Sensitivity to Fas-mediated apoptosis was evaluated using an anti-Fas mAb to trigger the Fas signal and MTT assay.

**RESULTS.** Estrogen and/or progesterone-withdrawal induced apoptosis of endometrial cells (48% and 43% respectively, $p<0.05$). At the same time, hormonal withdrawal increased Fas and FasL expression as determined by RT-PCR and Western blot analysis. Treatment of the endometrial cells with an anti-Fas antibody further increases apoptosis after hormonal removal (69%), suggesting that the Fas pathway is functional in these cells. Furthermore, application of anti-FasL antibody prior to hormonal withdrawal prevents cellular death.

**DISCUSSION.** Apoptosis in reproductive tissues is a physiological mechanism that eliminates differentiated, senescent, or dysfunctional cells. The cyclic changes of estrogen and progesterone promote a clean up of the endometrium preventing the accumulation of differentiated/mutated cells. In the present study we show that sex-hormone withdrawal represents the signal for apoptosis, in part via the Fas/FasL system. These results underscore the importance of sex-hormone cyclicity for providing a protective effect against neoplastic transformation in the endometrium. It may serve as a basis for preferring administration of HRT in a sequential combined mode during the menopause.

**ACKNOWLEDGEMENT.** This work was supported by National Institutes of Health Grant R01 HD37137 (to G.M.).

**REFERENCES.**

1. Mor, G., Gutierrez, L., Eliza, M., Kahyaoglu, F., and Arici, A. (1998) Am. J. Reprod. Immunol. 40, 89-95
2. Song, J., Sapi, E., Brown, W., et al. (2000) J. Clin. Invest. 106, 1209-1224
3. Mor, G., Kohen, F., Munoz, A., et al. (2000) J. Steroid Biochem. Mol. Biol. 73, 185-194
